# Anxiety and perceived social support as moderators of cognitive and emotional well-being in populations affected by COVID-19

**DOI:** 10.3389/fpubh.2025.1562894

**Published:** 2025-07-24

**Authors:** Jonathan Zegarra-Valdivia, Brenda Chino-Vilca, Leandro Pérez-Fernández, Milagros Casimiro-Arana, Harold Arana-Nombera, Viviana Nayelli Gallegos-Manayay, María del Rosario Oliva-Piscoya, Reyna Alamo-Medina, Eduardo Abanto-Saldaña, Nobuko Vásquez-Zuñe, Lisseth Detquizan Pérez, Diana Gutierrez-Flores, Leslie Lozada Tantarico, Naydelin Hernández, María Celinda Cruz-Ordinola, Carmen Paredes-Manrique

**Affiliations:** ^1^Facultad de ciencias de la salud, Universidad Señor de Sipán, Chiclayo, Peru; ^2^Laboratory of Neurobiology of Insulin Peptides, Achucarro Basque Center for Neuroscience, Leioa, Bizkaia, Spain; ^3^Department of Psychology, Faculty of Health Sciences, University of Deusto, Bilbao, Bizkaia, Spain; ^4^Facultad de psicología, Universidad Tecnológica del Perú, Lima, Peru

**Keywords:** COVID-19, anxiety, social cognition, perceived social support, cognitive performance

## Abstract

**Introduction:**

The COVID-19 pandemic has raised significant concerns about its long-term impact on cognitive and emotional functioning. This study explores the role of anxiety and social variables in shaping cognitive performance among individuals with a history of COVID-19 infection.

**Methods:**

This retrospective cross-sectional study included 227 Peruvian adults, classified into control, acute phase, and hyperinflammatory phase groups based on COVID-19 symptomatology. Cognitive performance was assessed using the Addenbrooke’s Cognitive Examination (ACE), focusing on global cognition. Anxiety levels, social cognition, and perceived social support were also measured. Moderation analyses were conducted to explore whether anxiety moderated the relationship between social cognition and perceived support, and whether support moderated the relationship between age and cognition.

**Results:**

Individuals with a history of COVID-19, particularly those in the acute and hyperinflammatory phases, showed significantly lower ACE scores than controls. Anxiety moderated the relationship between social cognition and perceived social support, with stronger associations at higher anxiety levels. Additionally, perceived support moderated the effect of age on cognitive performance, especially in individuals with low to moderate support.

**Discussion:**

These findings highlight the complex interplay between psychological and social factors in cognitive functioning following COVID-19. Understanding these relationships is crucial for developing integrated interventions that address cognitive and psychosocial recovery in affected populations.

## 1 Introduction

The emergence of the COVID-19 pandemic in late 2019 led public health systems into an era of unprecedented global health challenges, extending beyond the immediate threat of viral infection to encompass profound societal and psychological repercussions (1–3). As governments worldwide implemented stringent measures to curb the spread of the virus, the resultant shift in daily life precipitated a myriad of mental health concerns due to significantly disrupted social norms and routines (4). The pandemic’s far-reaching impact necessitates comprehensively examining its effects on mental well-being. Different studies report a prevalence ranging from 24.1 to 50% for depression, anxiety, stress, post-traumatic stress disorder (PTSD), and sleep disturbances (5). These outcomes were not only psychological but were also reflected in increased use of mental health services and diagnoses of panic disorders and acute stress reactions (4).

Lockdowns and social distancing measures led to isolation from support networks, unemployment, and persistent uncertainty (6). These restrictions, while crucial in mitigating viral transmission, also intensified anxiety and depression, particularly due to reduced community cohesion and decreased social interaction (7, 8). Neighborhood cohesion and a sense of community played a critical buffering role against psychological distress, while social connection, although protective, also influenced risk perception and protective behaviors (9). Additionally, contact with familiar individuals in private settings lowered risk perception, potentially increasing vulnerability to contagion (8).

The mental health consequences of lockdowns were multifaceted, affecting individuals across diverse demographics differently (10, 11). Vulnerable groups, including the older adults, those with pre-existing mental health conditions, and people facing socioeconomic hardship, experienced exacerbated symptoms (12–14). The closure of schools and the transition to remote learning posed additional challenges for children and adolescents, potentially impacting emotional stability and cognitive development (15–19). In the Peruvian context, adults with pre-existing mental health conditions, women, young people, and low-income individuals reported worsening depressive symptoms, exacerbated by loneliness and social isolation (20, 21). Older adults also reported increased anxiety, depression, and fatigue, partly due to restricted access to healthcare services (22).

In addition to psychological distress, lockdowns limited access to mental health services (23, 24). Restrictions on in-person consultations and the overwhelming demand for psychological support further strained health systems, exposing gaps in infrastructure and underscoring the importance of adaptable solutions, such as telemedicine. Meanwhile, the intersection of mental health and cognitive functioning during the pandemic has gained growing attention. Chronic stress and anxiety impair cognitive processes, including attention, memory, and executive functioning (25–27). A recent meta-analysis indicates that nearly one in four individuals with long COVID experience mental health issues (28). Moreover, cohort studies confirm sustained risk of psychiatric and neuropsychiatric disorders, particularly in middle-aged and older adults (29).

Infection with COVID-19 has also been linked to persistent cognitive effects, including deficits in memory and attention (30). Recent research highlights that these impairments may stem from altered brain functional connectivity, which correlates with reduced information processing efficiency and performance (31). The sustained psychological burden and neurobiological effects imposed by the pandemic may disrupt individuals’ cognitive functioning long after recovery (32–36).

In Peru, few studies have addressed these cognitive consequences in depth. Although recent work has begun to describe post-COVID cognitive effects in Peruvian adults (27, 32), the mechanisms underlying these impairments, especially involving emotional and social moderators, remain poorly understood. Furthermore, studies show that loneliness increased significantly during the pandemic, particularly emotional loneliness, and has been associated with increased symptoms of depression and anxiety (37, 38). Social isolation and the reduction of meaningful confidant interactions were key contributors, while electronic communication reduced loneliness and depressive symptoms (39).

Social cognition and perceived social support are particularly relevant in this context. Social cognition, which encompasses recognizing emotions and understanding others’ mental states, plays a central role in interpersonal interactions and mental health. It may be especially vulnerable to the effects of isolation and stress. Anxiety, one of the most prevalent psychological responses to the pandemic, has been associated with altered social processing and impaired emotional regulation. Furthermore, perceived social support has been shown to buffer stress’s adverse effects and moderate age-related cognitive decline (9). However, the interactions among social cognition, anxiety, social support, and cognitive performance in individuals affected by COVID-19 remain largely unexplored.

Therefore, the present study aims to examine the relationships among cognitive performance, social cognition, anxiety, and perceived social support in adults with a history of COVID-19. Specifically, we explore whether anxiety moderates the association between social cognition and perceived social support, and whether perceived social support moderates the relationship between age and cognitive performance. This approach is informed by psychosocial frameworks that posit emotional and social factors as key modulators of cognitive function (9, 31, 40).

Based on prior literature, we hypothesize that (a) individuals with a history of COVID-19 will exhibit lower cognitive performance than controls; (b) anxiety will moderate the relationship between social cognition and perceived social support, such that the association is stronger at higher anxiety levels; and (c) perceived social support will moderate the association between age and cognitive performance, particularly in individuals with lower support levels.

By integrating emotional and social variables, this study contributes to a more comprehensive understanding of post-COVID neurocognitive outcomes. These findings may inform the development of rehabilitation strategies that incorporate both psychosocial and cognitive components and highlight the importance of support systems in mitigating the long-term effects of COVID-19 on mental and cognitive health.

## 2 Methods

### 2.1 Design

A retrospective cross-sectional study was conducted with a cohort of participants with a history of COVID-19, along with thoroughly evaluated controls. The detailed characteristics of this study are described elsewhere (32). The present work uses data from the study conducted between 2022 and 2024, which collected cognitive, neuropsychiatric, and sociodemographic information from Peruvian participants.

### 2.2 Participants

Three hundred fifty-two participants were recruited through advertisements and assessments carried out on adults by our research team in various public institutions, including colleges and hospitals within Chiclayo, one major city in northern Peru. This strategic approach enabled us to effectively reach and engage a diverse group of individuals for our study. The inclusion criteria were defined separately for the COVID-19 and control groups as follows:

For the COVID-19 group:

1) A confirmed diagnosis of COVID-19 based on PCR or antigen testing.
2) Documented clinical symptoms consistent with COVID-19, as recorded in medical history.
3) A follow-up medical evaluation reconfirming the infection and symptomatology.
4) No prior history of brain injury, neurological disorders, or psychiatric treatment.
5) Completion of the full cognitive assessment protocol.
6) Only a single episode of COVID-19 infection (participants with multiple infections were excluded).
7) Provided informed and voluntary consent for participation.

For the control group:

1) No history or diagnosis of COVID-19.
2) A negative PCR or antigen test result for SARS-CoV-2.
3) No prior history of brain injury, neurological disorders, or psychiatric treatment.
4) Completion of the full cognitive assessment protocol.
5) Provided informed and voluntary consent for participation.

In addition, exclusion criteria such as (1) absence of PCR/antigen tests or doubtful diagnoses and (2) withdrawal by incomplete evaluation and cognitive assessment should also be considered. The subjects gave written informed consent to participate in the study and underwent a neuropsychological evaluation to measure the cognitive status of the sample. Lastly, the present study included data from 227 participants who had available and valid data from the original cohort. A detailed list of the sample characteristics can be found in Table 1.

**Table 1:** Sociodemographic and clinical characteristics of the sample.

	All (*n* = 227)	CG (*n* = 70)	AP (*n* = 123)	HP (*n* = 34)	ANOVA	*p*-valor	*Post-hoc*		
	Mean ± SD	Mean ± SD	Mean ± SD	Mean ± SD			CG vs AP	CG vs HP	AP vs HP
Age	44.16 ± 18.38	49.64 ± 19.13	41.52 ± 17.70	42.44 ± 17.22	4.678	0.01	0.09	n.s.	n.s.
Female (%)	62.6	67.1	56.9	73.5			n.s.		
Years of education	6.05 ± 2.06	5.857 ± 2.09	6.211 ± 1.92	5.852 ± 2.43	0.843	0.432	n.s.	n.s.	n.s.
Elapsed time-COVID*	453.5 ± 368.5	–	586.2 ± 298.3	576.9 ± 379.2			<0.01	<0.01	n.s.
DASS-21	6.550 ± 7.548	4.842 ± 7.101	7.186 ± 7.661	7.764 ± 7.651	2.709	0.069	n.s.	n.s.	n.s.
DASS-DEP	1.687 ± 2.626	1.185 ± 2.195	1.959 ± 2.892	1.735 ± 2.326	1.959	0.143	n.s.	n.s.	n.s.
DASS-ANX	2.026 ± 2.638	1.614 ± 2.622	2.178 ± 2.608	2.323 ± 2.760	1.278	0.281	n.s.	n.s.	n.s.
DASS-STR	2.845 ± 3.007	2.042 ± 2.840	3.048 ± 2.950	3.764 ± 3.238	4.498	0.012	n.s.	0.018	n.s.
Lubben	15.82 ± 6.755	14.67 ± 6.562	16.86 ± 7.220	14.41 ± 4.566	3.302	0.039	n.s.	n.s.	n.s.
UCLA	35.21 ± 8.86	33.13 ± 8.71	35.64 ± 9.14	33.53 ± 6.89	2.135	0.124	n.s.	n.s.	n.s.
Mini-Sea	11.89 ± 2.064	11.31 ± 2.271	12.18 ± 1.973	12.01 ± 1.720	4.171	0.017	0.014	n.s.	n.s.
ACE	88.56 ± 8.989	93.24 ± 3.465	86.79 ± 9.865	85.35 ± 9.987	15.875	<0.01	<0.01	<0.01	n.s.

Data are presented as mean ± standard deviation (SD). ANOVA was employed to assess group differences, with *post-hoc* pairwise comparisons conducted where applicable. Group definitions are as follows: CG (controls), AP (acute response to COVID-19), and HP (hyperinflammatory response to COVID-19). The variables used into the analyses are age (years), sex (female %), education (years), duration of elapsed time between infection (from COVID-19 diagnosis) and the evaluation (Elapsed time-Covid), Depression Anxiety Stress Scales (DASS-21), depression subscale score (DASS-DEP), anxiety subscale score (DASS-ANX), stress subscale score (DASS-STR), perceived social support (Lubben), perceived loneliness (UCLA: Loneliness Scale—Revised Version), social cognition (Mini-Sea: Mini Social Cognition and Emotional Assessment), and global cognitive status (ACE: Addenbrooke’s Cognitive Examination-Peruvian Version). Statistical significance is indicated as *p < 0.05; **p < 0.01, while “n.s.” denotes non-significant results.

### 2.3 Instruments

**Depression Anxiety Stress Scales-21 (DASS-21)**. Brief self-report tool to evaluate depression, anxiety, and stress levels. It includes 21 items evenly distributed across three subscales. Participants rated their experience over the past week on a 4-point scale from 0 (did not apply to me at all) to 3 (applied very much or most of the time). Each emotional state (depression, anxiety, and stress) is evaluated with seven items. Subscale scores range from 0 to 21; higher scores indicate greater symptom severity in the respective dimensions. Validated across various contexts (41–43), the DASS-21 demonstrates excellent internal consistency and reliability, making it a trusted instrument in clinical and research settings. Its concise format and straightforward administration make it a practical choice for quickly identifying individuals who may require psychological support and for guiding the development of targeted mental health interventions.

**Lubben Social Network Scale (Lubben)**. The Lubben Social Network Scale (LSNS) assesses social networks and screens for social isolation, particularly among older adults. The scale measures the number and frequency of social contacts with family and friends and the perceived support from these contacts. LSNS has been validated in various populations and settings, demonstrating good psychometric properties (44, 45). The LSNS-6 consists of six items, divided into two subscales: Family and Friends, each with three items. Each item is scored from 0 (none) to 5 (nine or more people), yielding a total score ranging from 0 to 30. Scores below 12 indicate risk of social isolation, while higher scores reflect stronger perceived support. In this study, we used the total score. It is a practical tool for clinical and research settings, providing valuable insights into the social dimensions of health and well-being in older populations.

**UCLA Loneliness Scale—Revised Version**. This 20-item self-report instrument assesses the subjective perception of loneliness and dissatisfaction with social relationships. It captures emotional loneliness (the absence of intimate relationships) and social loneliness (lack of integration into a broader social network). Each item is rated on a 4-point Likert scale ranging from 1 (never) to 4 (often), yielding a total score from 20 to 80. Higher scores reflect greater perceived loneliness. The total score was used in all analyses.

**Mini Social Cognition and Emotional Assessment (Mini-Sea)**. A brief neuropsychological tool to evaluate social cognition deficits, particularly in recognizing emotions and understanding others’ mental states (46). It comprises two subtests: the Facial Emotion Recognition (FER) test, assessing the ability to identify emotions from facial expressions, and the Faux Pas Recognition Test (FPRT), evaluating theory of mind through the detection of social blunders in short narratives. It has been adapted to Spanish by Henriques et al. (47). The total Mini-Sea score ranges from 0 to 30, with higher scores indicating better social cognitive performance. In this study, the total score was used for analysis.

**Addenbrooke’s Cognitive Examination (Peruvian Version)**. Participants were screened using the ACE (Peruvian Version) to evaluate their global cognitive status. It assesses six domains: attention and orientation, memory, fluency, language, and visuospatial abilities, providing a total score out of 100. The ACE-P is utilized clinically and in research for the detection and monitoring of cognitive impairments such as dementia. It has a good consistency and validity in the Peruvian population (48, 49).

### 2.4 Statistical analysis

The cohort was divided into two groups: healthy controls with no reported history of COVID-19 infection and participants who reported having been infected with COVID-19, confirmed by q-PCR or antigen test. These subjects were stratified according to the number of days reported with symptoms in the acute phase (AP), between one and 14 days, or hyperinflammatory phase (HP) more than 14 days (50). To ensure the quality and reliability of the data, we conducted several preliminary analyses before the formal analyses. First, we use descriptive statistics to assess the frequencies, percentages, central tendency, and dispersion measures. Parametric and non-parametric contrast tests (Chi2, Kruskal Wallis H test) were used depending on the normality (checked using Kolmogorov—Smirnov test) and homogeneity of variances (Levene test).

Furthermore, age and the duration of elapses between infection (from COVID-19 diagnosis) and the cognitive evaluation were evaluated using a one-way ANOVA, finding differences between the ages but not in the elapsed duration of the patients with COVID history. Considering this effect, the second step assessed the differences between variables selected using an ANCOVA analysis with age and education as covariates in all comparisons, adjusting the results for multiple comparisons (Bonferroni correction).

The next step of the analysis was to evaluate the moderation effect of the variables. Moderation analysis investigated the conditions that facilitate, enhance, or inhibit the impact of one variable called a moderator over the effect of X on Y. So, the final model was constructed considering two steps. Step one is to evaluate the moderator effect of anxiety (DASS-ANX) on the relationship between social and emotional recognition (Mini-Sea) and perceived social support (Lubben). The adjusted model includes age, sample division (Group), and education as covariates, and the second step evaluates if the perception of social support moderates the association between age and general cognitive performance (see Table 2). The computed tool used for this purpose is Process Macro, an extension for SPSS. Both steps included an additional analysis of simple slopes to select the value or values of the moderator W, calculating the effect of X on Y at the value, which was called the Johnson-Neyman technique. It helps us identify the specific values of a moderator variable at which the effect of the focal predictor on the outcome variable becomes statistically significant. Statistical analysis was performed with SPSS version 24 (SPSS, Inc., Armonk, NY, United States). Significant results are reported with *p* < 0.05* and *p* < 0.01**.

**Table 2:** Model construction: role of education, social cognition, and social network.

	Coeff.	SE	t	*p*
Model 1				
Social Cognition (X)	−0.36	0.309	−1.168	0.244
Anxiety (W)	−3.87	1.15	−3.35	0.0009
Social Cognition x Anxiety (XW)	0.334	0.0917	3.678	0.0003
Age (C1)	0.363	0.239	1.517	0.131
Education (C1)	0.052	0.029	1.816	0.071
Group (C1)	0.787	0.678	1.161	0.247
Constant	14.287	4.603	3.103	0.002
R^2^ = 0.107; MSE = 41.893				
*F*(6, 220) = 4.370; *p* < 0.001				
Model 2				
Age (X)	−0.389	0.127	−3.052	0.0028
Perceived Social Support (W)	−0.1914	0.2,532	−0.7,561	0.4,511
Age x Perceived Social Support (XW)	0.0164	0.0072	2.276	0.0246
Constant	94.57	4.577	20.66	<0.001
R^2^ = 0.132; MSE = 86.585				
*F*(3, 119) = 6.0452; *p* < 0.001				

This table provides the coefficient (Coeff.) and *p* values for each variables included in the model construction process. Model 1 described variables such social cognition (Mini-Sea; X), Anxiety (DASS-ANS; W), social cognition x anxiety (XW), and covariables such as age, education (years), and group (sample division). Model 1: F (6, 220) = 4.370; *p* < 0.001. Model 2: Includes age (X), perceived social support (W), and age x perceived social support (XW), F (3, 119) = 6.0452; *p* < 0.001. The use of education as a covariable did not show any significant result in the Model 2.

## 3 Results

The sample’s mean age was 44.16 years old (± 18.38), and 62.6% of the participants were females. Regarding DASS-21, the mean score for the subject with a history of Covid infection was higher (AP: 7.19 ± 7.67; HP: 7.76 ± 7.65) than the control group (CG: 4.82 ± 7.101); however, the differences between the groups were no significant for the total score, depression (DASS-DEP), and anxiety (DASS-ANX). The stress subscale shows differences between AP and the control group (*p* = 0.018). Lubben scores show a similar pattern across the group without significant results. Finally, regarding neuropsychological performance, the ACE score was higher in the control group (93.24 ± 3.47), showing significant differences compared with the AP (86.79 ± 9.87) and the HP group (85.35 ± 9.99), highlighting the impact of COVID-19 infection on cognitive performance. See more details on Table 1.

Table 3 reports the relationship between sociodemographic data, social support, psychopathological symptoms, and cognitive performance according to each group. Across the sample, age was negatively associated with education, psychopathological symptoms, and loneliness. This means that age is related to fewer years of schooling (Coeff. –0.34; *p* < 0.01), fewer psychopathological symptoms (Coeff. –0.29; *p* < 0.01), and increased perception of loneliness (Coeff. –0.35; *p* < 0.01). Age is also associated with decreased Mini-Sea scores (Coeff. –0.41; *p* < 0.01). In the CG, age is significantly associated with lower levels of schooling (Coeff. –0.51; *p* < 0.01) fewer psychopathological symptoms (DASS-21: Coeff. –0.45; *p* < 0.01; DASS-DEP: Coeff. –0.26; *p* < 0.05; DASS-ANX: Coeff. –0.17; *p* < 0.05; DASS-STR: Coeff. –0.39; *p* < 0.01), decreased Mini-Sea scores (Coeff. –0.54; *p* < 0.01), and decreased global cognitive performance (ACE: Coeff. –0.24; *p* < 0.05). Schooling, in turn, is positively associated with higher perception of loneliness (UCLA: Coeff. 0.32; *p* < 0.01), and higher Mini-Sea (Coeff. 0.32; *p* < 0.01).

**Table 3:** Correlation analysis of sociodemographic and psychological variables.

		Age	Education (years)	Lubben	DASS-21	DASS-DEP	DASS-ANX	DASS-STR	UCLA	Mini-Sea	ACE
All sample	Age	1.000									
	Education (years)	−0.341^**^	1.000								
	Lubben	n.s.	n.s.	1.000							
	DASS-21	−0.289^**^	n.s.	n.s.	1.000						
	DASS-DEP	−0.174^**^	n.s.	n.s.	0.799^**^	1.000					
	DASS-ANX	−0.166^*^	n.s.	0.157^*^	0.842^**^	0.620^**^	1.000				
	DASS-STR	−0.337^**^	n.s.	n.s.	0.875^**^	0.567^**^	0.592^**^	1.000			
	UCLA	−0.345^**^	n.s.	−0.204^**^	0.546^**^	0.499^**^	0.442^**^	0.463^**^	1.000		
	Mini-Sea	−0.408^**^	0.224^**^	n.s.	0.192^**^	n.s.	0.199^**^	0.135^*^	0.171^**^	1.000	
	ACE	n.s.	n.s.	n.s.	n.s.	n.s.	n.s.	n.s.	n.s.	0.264^**^	1.000
CG	Age	1.000									
	Education (years)	−0.512^**^	1.000								
	Lubben	n.s.	n.s.	1.000							
	DASS-21	−0.451^**^	n.s.	n.s.	1.000						
	DASS-DEP	−0.256^*^	n.s.	n.s.	0.724^**^	1.000					
	DASS-ANX	−0.487^**^	n.s.	n.s.	0.861^**^	0.508^**^	1.000				
	DASS-STR	−0.394^**^	n.s.	n.s.	0.885^**^	0.526^**^	0.638^**^	1.000			
	UCLA	−0.504^**^	0.316^**^	−0.317^**^	0.377^**^	0.275^*^	0.332^**^	0.344^**^	1.000		
	Mini-Sea	−0.540^**^	0.324^**^	n.s.	0.346^**^	n.s.	0.340^**^	0.251^*^	0.280^*^	1.000	
	ACE	−0.242^*^	n.s.	n.s.	n.s.	n.s.	n.s.	n.s.	n.s.	0.378^**^	1.000
AP	Age	1.000									
	Education (years)	−0.194^*^	1.000								
	Lubben	0.209^*^	0.051	1.000							
	DASS-21	−0.177^*^	n.s.	n.s.	1.000						
	DASS-DEP	n.s.	n.s.	n.s.	0.846^**^	1.000					
	DASS-ANX	n.s.	−0.205^*^	n.s.	0.824^**^	0.683^**^	1.000				
	DASS-STR	−0.317^**^	n.s.	n.s.	0.849^**^	0.577^**^	0.519^**^	1.000			
	UCLA	−0.275^**^	n.s.	−0.179^*^	0.650^**^	0.621^**^	0.504^**^	0.493^**^	1.000		
	Mini-Sea	−0.281^**^	n.s.	0.349^**^	n.s.	n.s.	0.230^*^	n.s.	n.s.	1.000	
	ACE	n.s.	n.s.	0.256^**^	n.s.	n.s.	0.195^*^	n.s.	n.s.	0.317^**^	1.000
HP	Age	1.000									
	Education (years)	−0.497^**^	1.000								
	Lubben	n.s.	n.s.	1.000							
	DASS-21	n.s.	n.s.	0.401^*^	1.000						
	DASS-DEP	n.s.	n.s.	n.s.	0.756^**^	1.000					
	DASS-ANX	n.s.	−0.429^*^	0.359^*^	0.861^**^	0.562^**^	1.000				
	DASS-STR	n.s.	n.s.	0.341^*^	0.944^**^	0.619^**^	0.751^**^	1.000			
	UCLA	n.s.	n.s.	n.s.	0.502^**^	0.451^**^	0.444^**^	0.566^**^	1.000		
	Mini-Sea	−0.347^*^	0.559^**^	n.s.	n.s.	n.s.	n.s.	n.s.	n.s.	1.000	
	ACE	n.s.	n.s.	n.s.	n.s.	n.s.	n.s.	n.s.	n.s.	0.343^*^	1.000

Data are presented as Pearson correlation coefficients (r). The variables used into the analyses are age (years). sex (female %), education (years), score of depression Anxiety Stress Scales (DASS-21), perceived social support (Lubben), perceived loneliness (UCLA: Loneliness Scale—Revised Version), social cognition (Mini-Sea: Mini Social Cognition and Emotional Assessment), and global cognitive status (ACE: Addenbrooke’s Cognitive Examination-Peruvian Version). Statistical significance is indicated as *p < 0.05; **p < 0.01, and “n.s.” denotes non-significant results.

In the AP subgroup, age is negatively associated with perceived loneliness (UCLA, Coeff. –0.28; *p* < 0.01), while Mini-Sea scores are positively correlated with social connectedness (Lubben; Coeff. 0.35; *p* < 0.01). For the HP subgroup, schooling shows a strong positive association with Mini-Sea scores (Coeff. 0.56; *p* < 0.01), and psychopathological symptoms (DASS-21) correlate positively with both social connectedness (Lubben; Coeff. 0.34; *p* < 0.05) and perceived loneliness (UCLA; Coeff. 0.50; *p* < 0.01). These results underscore distinct relational dynamics across groups, reflecting the potential influence of COVID-19 symptomatology. More detailed information about the correlation between each group can be seen in Table 3.

**Figure 1:**
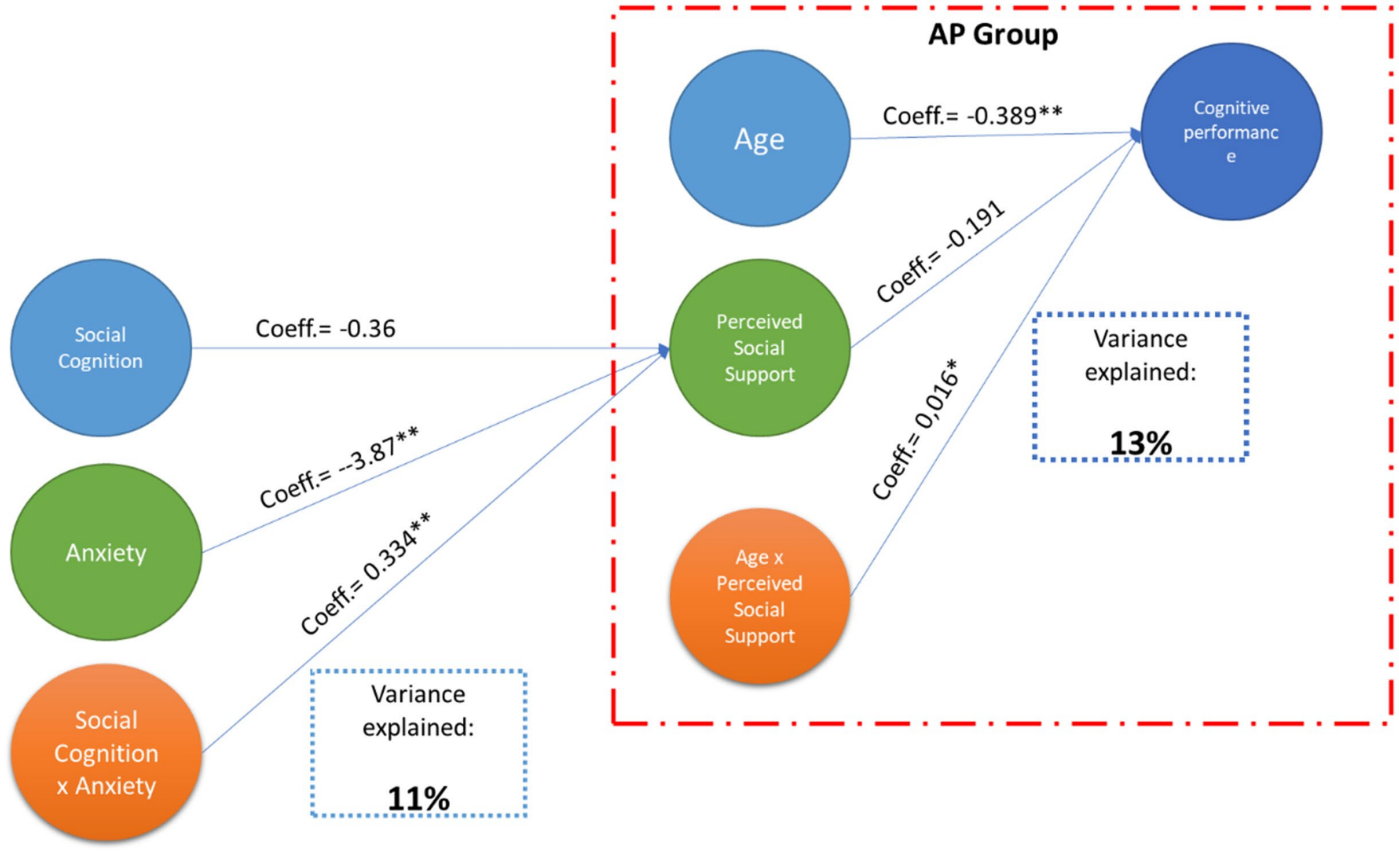
A conceptual model for social and cognitive outcomes in COVID-19. The diagram illustrates the conceptual model and the variance for cognitive performance explained in the second model. Blue arrows represent direct effects. The red dashed outline emphasizes that the moderation effect appears only in the AP group. Perceived social support moderates the relationship between age and cognitive performance, with this relationship being significant only in people who have suffered from COVID in the acute phase.

The proposed model was constructed in two steps. The first step was to evaluate the moderator effect of anxiety (DASS-ANX) on the relationship between social and emotional recognition (Mini-Sea) and perceived social support (Lubben scale). The adjusted model, including age, sample classification (Group), and education as covariables, explained 11% of the variance, as shown in Figure 1. Step 1, being the moderation statistically significant, *F* (6, 220) = 4.370; *p* < 0.001. Table 2 shows the information about the parameters included in the model. The results suggest that the effect of social and emotional recognition in the perception of social support is statistically significant, especially between people with over 2.42 points of anxiety (B anxiety = 2.42 = 0.454; *p* = 0.05).

A second step was to evaluate if the perception of social support moderates the association between age and general cognitive performance. The primary approximation was to probe the model on all the samples. However, the results were not significant. Then, the model was evaluated in each group, showing significant results only in the AP group, *F* (3, 119) = 6.0452; *p* < 0.001 (See Figure 1). The adjusted model in the AP group explained 13% of the variance; it means that the effect of age on cognitive performance is statistically significant only in scores below 17.85 in the perception of social support (B Lubben = 17.85 = −0.097; *p* = 0.05).

**Figure 2:**
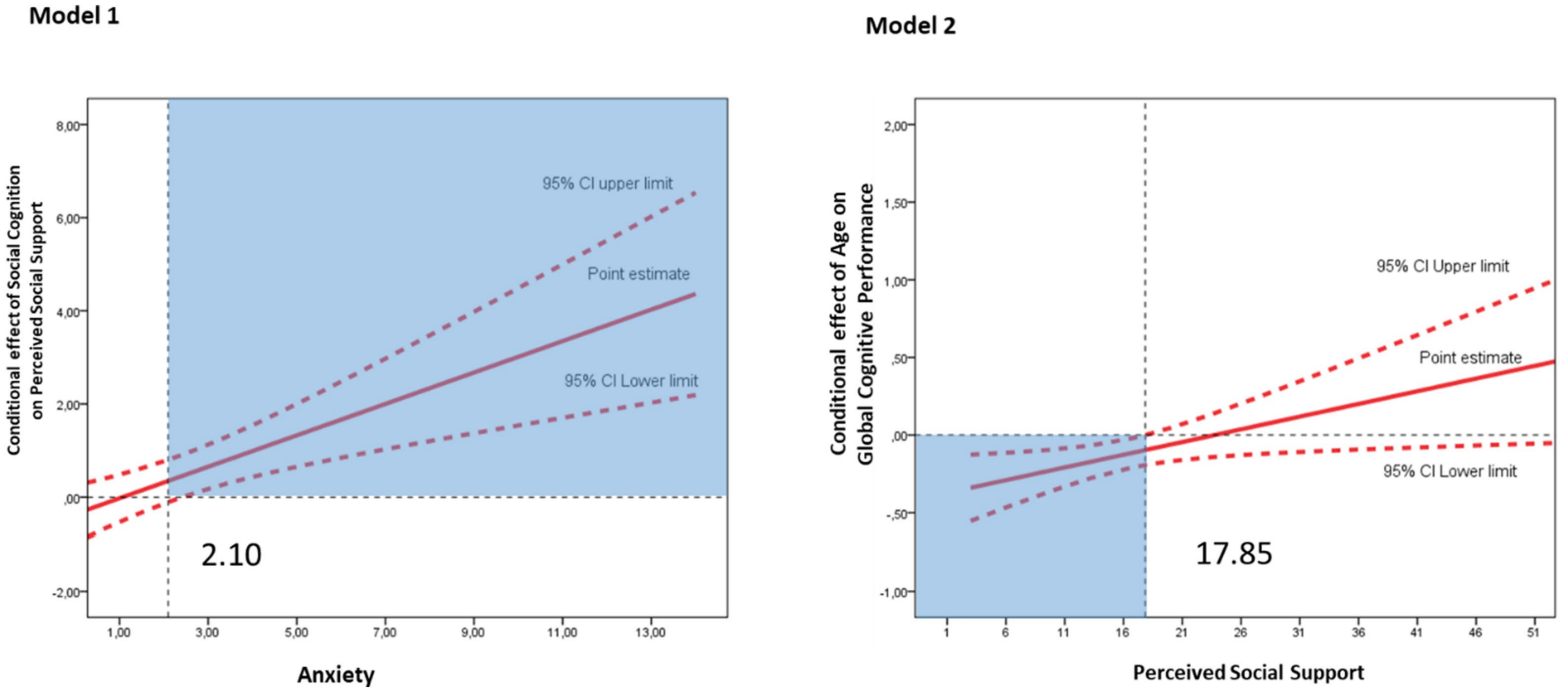
Interaction effects of anxiety and perceived social support on social and cognitive outcomes. The plots display the interaction effects analyzed in the regression models. Solid red lines represent the point estimates, while dashed red lines denote the 95% confidence intervals (CI). The blue-shaded area in the plots marks the moderator values where the interaction effect becomes statistically significant.

Figure 2 presents interaction plots illustrating the moderating effects within our proposed models. In model 1, the anxiety (DASS-ANX) moderates the effect of social cognition (Mini-Sea) on the perceived social support (Lubben), as indicated by the positive slope of the point estimate and the confidence intervals that exclude zero, mainly present in the anxiety score of 2.10. This suggests that individuals with anxiety rely more on their ability to recognize social and emotional cues to perceive social support. In the right panel, the interaction plot examines how perceived social support (Lubben) moderates the association between age and cognitive performance. The results show that the effect of age on cognitive performance is significant only at lower levels of social support, with significance beginning at a Lubben score of 17.85. Together, these findings highlight the critical role of anxiety and perceived social support as moderators in shaping the relationships between social–emotional variables, age, and cognitive outcomes.

## 4 Discussion

The COVID-19 pandemic has profoundly impacted mental health and social dynamics. Several studies have shown intensified anxiety during the pandemic and how it impairs emotional regulation and social interactions due to lockdown (51), while robust social support networks play a protective role in mitigating stress, anxiety, depression (52, 53), and cognitive decline (54). Despite this, the specific processes by which anxiety and social support interact to influence cognitive and social outcomes in people infected with COVID-19 remain poorly understood. This study aimed to address this gap by evaluating the moderating effects of anxiety and perceived social support on key neurocognitive and psychosocial relationships, particularly within the context of individuals affected by COVID-19.

The findings of this study provide crucial insights into the neurocognitive and psychosocial effects of COVID-19, particularly highlighting the moderating roles of anxiety and perceived social support. Firstly, the findings underscore a significant impact of COVID-19 infection on general cognitive functioning, as assessed by the ACE. Participants in both the AP and HP groups exhibited notably lower cognitive performance compared to controls. These results align with a growing body of evidence highlighting the detrimental effects of COVID-19 infection on cognitive processes, reinforcing its potential role in exacerbating cognitive decline and neuropsychological impairments (55, 56).

Although no significant group differences were found for depression and anxiety scores at the time of evaluation, the acute phase group did show elevated stress levels. This could reflect residual psychological strain or long-term effects linked to infection severity and psychosocial disruption (57, 58). The relative normalization of anxiety and depression may relate to post-crisis emotional adaptation (59, 60), or inconsistent adherence to restrictions, as seen in the Peruvian context (61). Indeed, multiple longitudinal studies have shown a general decline in psychological distress after the initial peak of the COVID-19 pandemic (62–64), reinforcing the idea that emotional symptoms may decrease naturally over time for some individuals.

Through moderation analyses, we found that social cognition significantly influenced perceived social support in individuals with anxiety. This suggests that anxiety amplifies sensitivity to socio-emotional cues, as individuals rely more on facial expressions and social feedback to interpret their level of support (65–67). Prior literature confirms that anxiety alters attentional mechanisms, increasing the salience of emotional expressions and potentially biasing perceptions (66–68). Neuroimaging studies have demonstrated that individuals with elevated anxiety exhibit increased amygdala activation in response to social threat cues and rely more on regulatory control from prefrontal regions, such as the medial and dorsolateral prefrontal cortex, to process and manage emotional input (69). More recent reviews confirm that this prefrontal-amygdala interaction is central to the regulation of fear responses and emotional resilience, particularly in populations with heightened anxiety (70).

In addition, the perception of social support moderated the association between age and cognitive performance, but only in the acute-phase group and when support levels were low. These results support previous findings indicating that social networks can buffer age-related cognitive decline (54), but they also suggest a ceiling effect where high support no longer offers incremental benefit (71). Neurocognitive resilience may be reinforced by social presence and the quality and frequency of meaningful interactions (72).

## 5 Clinical implications

These findings carry meaningful clinical implications. Psychosocial variables, particularly anxiety and perceived support, modulate cognitive functioning in post-COVID populations. This underscores the need to integrate emotional and social assessments in cognitive rehabilitation programs. Interventions focused solely on cognitive retraining may be insufficient if anxiety and social disconnection persist. Strategies such as anxiety reduction, emotion recognition training, and structured social support enhancement (e.g., psychoeducation, family engagement, peer groups) could amplify cognitive recovery outcomes (72, 73). In resource-limited settings, where access to formal care is constrained, low-cost telepsychology interventions or community-led support programs may offer scalable solutions.

## 6 Strengths and contributions

This study offers several notable strengths. First, it addresses a novel and underexplored intersection between social cognition, anxiety, and cognitive performance in a post-COVID context, particularly in Latin America. Second, it distinguishes between individuals in the acute and hyperinflammatory phases, allowing for a more granular understanding of how symptom duration and severity impact neurocognition. Third, it integrates multiple psychological constructs: cognitive performance, emotional distress, social cognition, and perceived social support, offering a holistic view of mental functioning. Lastly, by employing moderation models, this study advances our understanding of the mechanisms underlying COVID-19’s impact on neuropsychological outcomes and highlights targets for intervention.

## 7 Limitations

It is essential to acknowledge the limitations of this study. The cross-sectional design limits the ability to infer causality, and reliance on self-reported measures may introduce bias. Therefore, future research should employ longitudinal designs and objective assessments to validate these findings. Additionally, although the models explained a modest proportion of the variance (11 and 13%, respectively), the significance of the results underscores the value of incorporating psychosocial variables in neurocognitive research. Further studies should investigate other potential moderators, such as cultural context or socioeconomic status, to better understand the complex interplay between cognition, social dynamics, and emotional well-being.

## 8 Conclusion

Our findings highlight the complex relationships between anxiety, social cognition, and social support, critical factors influencing individuals’ cognitive and emotional well-being following COVID-19. Lower cognitive performance among infected individuals, particularly those in more severe phases, reflects the lasting cognitive burden of the virus. The moderating roles of anxiety and support suggest that psychological and social variables can exacerbate or buffer these effects. Addressing these aspects is essential for improving long-term mental health outcomes. Future research should pursue longitudinal designs and explore integrative interventions that combine cognitive training with emotional and social rehabilitation. Public health policies should prioritize access to psychosocial care and support networks, particularly in vulnerable populations.

## Data Availability

Further inquiries about data available upon reasonable request can be directed to the corresponding author.
